# Transition from two-dimensional photonic crystals to dielectric metasurfaces in the optical diffraction with a fine structure

**DOI:** 10.1038/srep30773

**Published:** 2016-08-05

**Authors:** Mikhail V. Rybin, Kirill B. Samusev, Stanislav Yu. Lukashenko, Yuri S. Kivshar, Mikhail F. Limonov

**Affiliations:** 1 Ioffe Institute, St. Petersburg 194021, Russia; 2 ITMO University, Department of Photonics of Dielectrics and Semiconductors, St. Petersburg 197101, Russia; 3 ITMO University, Department of Nanophotonics and Metamaterials, St. Petersburg, 197101, Russia; 4 Institute for Analytical Instrumentation RAS, Department of Nanophotonics and Metamaterials, St. Petersburg 198095, Russia; 5 Australian National University, Nonlinear Physics Center, Canberra ACT 2601, Australia

## Abstract

We study experimentally a fine structure of the optical Laue diffraction from two-dimensional periodic photonic lattices. The periodic photonic lattices with the *C*_4*v*_ square symmetry, orthogonal *C*_2*v*_ symmetry, and hexagonal *C*_6*v*_ symmetry are composed of submicron dielectric elements fabricated by the direct laser writing technique. We observe surprisingly strong optical diffraction from a finite number of elements that provides an excellent tool to determine not only the symmetry but also exact number of particles in the finite-length structure and the sample shape. Using different samples with orthogonal *C*_2*v*_ symmetry and varying the lattice spacing, we observe experimentally a transition between the regime of multi-order diffraction, being typical for photonic crystals to the regime where only the zero-order diffraction can be observed, being is a clear fingerprint of dielectric metasurfaces characterized by effective parameters.

Diffraction of waves of different nature (e.g., X-rays, electrons, neutrons, photons, etc) is a common phenomenon underlying many experimental tools employed for the study of crystalline structures and the analysis of physical properties of ordered bulk materials^1^. Nowadays, diffraction of electrons is widely used to detect the number of stacking sheets of planar two-dimensional (2D) materials such as graphene^2^, carbon nanofilms^3^, transition metal dichalcogenides^4^, etc. For the three-dimensional (3D) photonic crystals, when the period of the spatial modulation of dielectric permittivity becomes comparable with the wavelength of light, the Bragg diffraction gives rise to the appearance of the bandgaps in the energy spectrum^5^,^6^,^7^. An instructive example is the analysis of optical Bragg diffraction from different opal-based photonic structures, including thin opal films^8^,^9^,^10^, bulk samples of synthetic opals^11^,^12^,^13^, and opal-based colloidal structures^14^,^15^. Opals are built up of quasi-spherical particles of amorphous silica *a*-SiO_2_, each of them having a rather hard shell and porous core^16^. Opals possess bandgaps in the visible range due to the typical size of the constitutive particles *a*-SiO_2_ of some hundreds of nanometers. This provides a unique way for the direct observation of angle-resolved diffraction patterns in the visible spectral range.

Thin photonic films composed of several layers have been studied by spectroscopic techniques. In particular, the results of optical studies of opal films composed of 6 layers are given in ref. 10. Also, diffraction patterns from woodpile films consisting of 20 layers were studied depending on the internal refractive index contrast Δ*n* in ref. 17. A detailed picture of a transformation of the optical diffraction patterns during the transition from thin opals films to 3D opal-based photonic crystals was studied in refs 7 and 10.

In spite of a large amount of experimental studies of periodic structures^18^,^19^,^20^,^21^, diffraction of light from two-dimensional planar photonic structures composed of just a single layer (the so-called metasurface) and several elements in the plane was not studied experimentally in detail, to the best of our knowledge. We emphasize that diffraction is a unique tool for the studies of optical properties of true two-dimensional structures because other methods, such as reflection and transmission spectroscopy, produce a very weak response from a single sub-micron layer. In contrast, here we demonstrate that the 2D structures composed of a finite number of sub-micron dielectric scatterers give rise to surprisingly strong intensity of optical diffraction that can be visible by a naked eye on a screen placed just behind the metasurface sample.

With the intention to further deepen our understanding of light scattering in periodic media, a number of challenging problems can be formulated: What are novel effects in optical diffraction from finite-size 2D photonic structures, beyond the well-known results of X-rays, neutrons, and photons diffraction from thin films and 3D structures? Can one obtain from the light scattering direct information about the number and spatial distribution of sub-micron particles? And finally the most intriguing question: is it possible to observe in optical diffraction a transition between 2D photonic films and metasurfaces? The study of metasurfaces has attracted much attention in recent years^22^,^23^ due to their many useful functionalities and potentially important applications, ranging from simple elements of flat optics for unusual beam steering^24^,^25^, high efficiency of planar sensing devices^26^ and other types of light control^27^,^28^ in both linear and nonlinear regimes^29^. We notice that a transition in light scattering regimes from photonic crystals to all-dielectric metamaterials and a corresponding phase diagram were studied in ref. 30.

In this study, we use a direct laser writing (DLW)^31^,^32^,^33^ technique to fabricate true 2D photonic structures or metasurfaces as periodic arrays of submicron dielectric particles or their inverted counterparts with the square *C*_4*v*_, orthogonal *C*_2*v*_ and hexagonal *C*_6*v*_ lattice symmetry. We study experimentally optical diffraction from fabricated direct and inverted finite-size 2D structures and observe directly (on a screen placed after the sample) a variety of diffraction patterns of exceeding beauty. The fine structure of the patterns allows detecting exact number of scatterers in any direction. Using a set of anisotropic samples with orthogonal *C*_2*v*_ lattice symmetry, we demonstrate both experimentally and theoretically a transition from multi-order diffraction regime which is characteristic for photonic crystals to the regime where only the zero-order diffraction was observed being a fingerprint of metasurfaces characterized by effective parameters.

## Results

### Sample fabrication

The problem of fabrication of 2D photonic structures of almost arbitrary shape can be solved with the recently developed DLW method. This technology is based on the nonlinear two-photon polymerization of a photosensitive material in the focus of a femtosecond laser beam. A high resolution of the technique is due to the intensity-threshold character of the polymerization process which occurs in a region with sizes significantly smaller than the size of the focused beam. This method makes it possible to create a dielectric structure with a transverse resolution below 100 nm^34^.

Using DLW technique we fabricated a variety of high-quality finite-size 2D photonic structures with submicron-scale features. To realize the DLW approach, we use the apparatus fabricated at the Laser Zentrum Hannover (Germany) and a train of femtosecond pulses centered at around 780 nm wavelength and at repetition frequency of 80 MHz (12.5 ns time between the adjacent pulses). These pulses are derived from a 50 fs TiF-100F laser (Avesta-Project, Russia). Photonic structures are fabricated by using a hybrid organic–inorganic material based on zirconium propoxide with an Irgacure 369 photoinitiator (Ciba Specialty Chemicals Inc., USA).

We fabricate both direct and inverted dielectric photonic structures as 2D periodic arrays of scatterers with the square *C*_4*v*_, orthogonal *C*_2*v*_ and hexagonal *C*_6*v*_ lattice symmetry. The samples with the square and orthogonal lattice symmetry were fabricated with the square or rectangular shape. The direct photonic structures are composed of dielectric particles with an ellipsoid-like shape (called ‘voxels’ in what follows) with a typical size of 100–300 nm in the surface plane. With “inverted photonic structure” we term a structured thin dielectric film with an array of holes.

**Figure 1:**
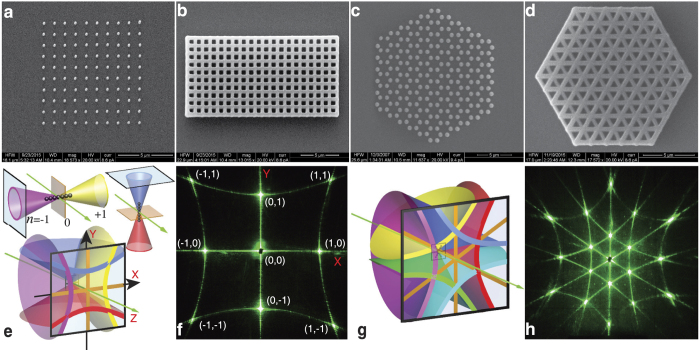
Examples of fabricated structures shown with SEM images: (**a**) direct square structure (10 × 10 voxels, *a*_1_ = *a*_2_ = 1 *μ*m), (**b**) inverted rectangular structure with square symmetry (10 × 20 holes, *a*_1_ = *a*_2_ = 1 *μ*m), (**c**) direct hexagonal structure (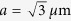), (**d**) inverted hexagonal structure (*a* = 1.5 *μ*m). (**e**) Schematic of the zero-order (*n* = 0) and first-order (*n* = ±1) Laue diffraction from the horizontally and vertically oriented chains of scatterers and from the structure with square symmetry composed of both types of chains in the case *a*_1_ = *a*_2_. Diffraction patterns on a flat screen are shown by thick lines. Scattered light is shown by different colors for clarity. (**f**) Experimental pattern for diffraction of monochromatic light (*λ* = 0.53 *μ*m) from an inverted structure of the square symmetry (100 × 100 holes, *a*_1_ = *a*_2_ = 1 *μ*m) observed on a flat screen positioned behind the sample. Main diffraction maxima are marked with the pairs of the diffraction indices (*n*_1_, *n*_2_). (**g**) Schematic of the zero- and first-order Laue diffraction from the hexagonal structure. (**h**) Experimental pattern for zero-, first- and second-order Laue diffraction of monochromatic light (*λ* = 0.53 *μ*m) from an inverted hexagonal structure (*a* = 1.5 *μ*m) observed on a flat screen positioned behind the sample.

The number of scatterers varied from 10 s to 10000 s. The lattice parameters varied in different samples in the range of 0.5 *μ*m  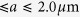. Examples of the images obtained from both direct and inverted photonic structures with different symmetries with a help of a scanning electron microscope are presented in Fig. 1.

### Fine structure in diffraction patterns

To analyze the fine structure of the diffraction patterns, we consider the scattering from one-dimensional (1D) linear chain of scatterers lying along **a**_1_, for this we set *N*_2_ = 1 in the Eq. (4) in Methods. The positions of the 1D diffraction strong maxima in the square of the structure factor modulus |*S*(**q**)|^2^ (called ‘the main maxima’ in what follows) corresponding to the condition of constructive interference are determined in the limit sin(**qa**_1_/2) → 0 that yields **qa**_1_ = (**k**_*i*_ − **k**_*s*_)**a**_1_ = 2*πn* and


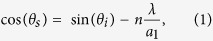


where *n* is integer that enumerates the diffraction order, *θ*_*i*_ is the incidence angle between the wave vector **k**_*i*_ and the normal vector to the chain, *θ*_*s*_ is the scattering angle between vectors **a**_1_ and **k**_*s*_. Now we analyze Eq. (1) to derive the conditions when the diffraction of the *n*-th order does not exist, that is the cosine in the left-hand side does not fit into the (−1, 1) interval


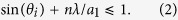


For the normal light incidence **k**_*i*_ ⋅ **a**_1_ = 0, the equation **k**_*s*_ ⋅ **a**_1_ = 2*πn* takes the form 
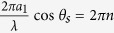
 that describes a family of cones pairs with the axes of symmetry coinciding with **a**_1_ and the apex angle of scattering is given by


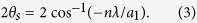


In the current study we mainly focus on the most important case of the normal incidence. The zero-order cone (*n* = 0) degenerates to the plane normal to **a**_1_ since the angle between **k**_*s*_ and **a**_1_ becomes *θ*_*s*_ = *π*/2. A pair of diffraction cones of the *n*-th order appears when *a*_1_ > *nλ*, but it is prohibited when the argument is outside of the arccosine domain |*nλ*/*a*_1_| > 1. We notice that the prohibited arcs related to the evanescent wave that do not affect far-field pattern. A chain of scatterers with the period of *a*_1_ = 1 *μ*m illuminated by a Nd laser with *λ* = 0.53 *μ*m scatters light in the zero-order plane, and the first-order (*n* = ±1) cones with *θ*_*s*_ = 58° [Fig. 1(e)]. In our experiments, a photonic structure with square lattice symmetry [Fig. 1(a,b)] can be considered as a structure composed of two sets of orthogonal chains along the *x*- and *y*-axes. In such case one can expect in the diffraction patterns two orthogonal planes and two families of orthogonal couples of cones, as shown in Fig. 1(e). The experimentally measured diffraction patterns from such structures with sufficiently large number of scatterers agree well with such a simple model [Fig. 1(f)]. Also we discuss the case of the oblique incidence, when the zero-order diffraction condition takes the form sin(*θ*_*i*_) + *λ*/*a*_1_ > 1 for the specified incident angle. For the case of an arbitrary incident angle, we have the following condition *λ*/*a*_1_ > 2. Here we notice that the current analysis is limited to the case of 2D structures that do not support guided modes. Otherwise, numerical methods can be exploited and the criteria of zero-order diffraction should be corrected by the effective refractive index.

**Figure 2:**
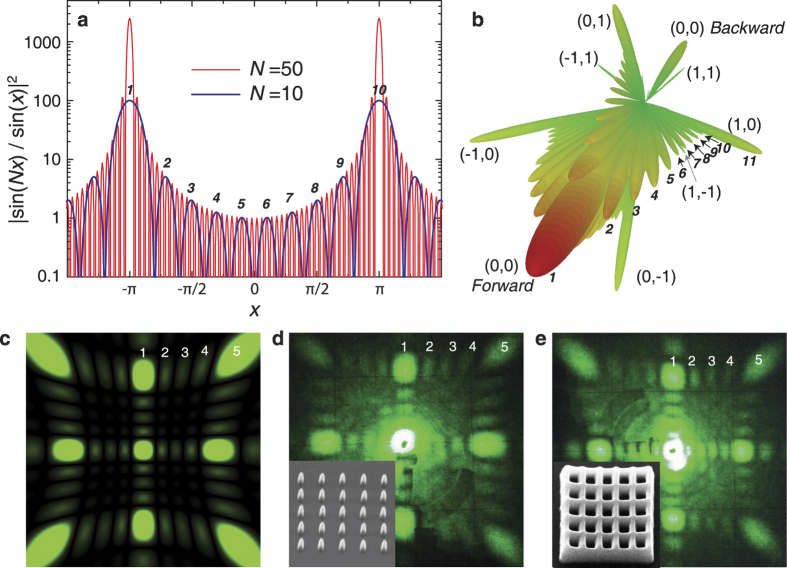
(**a**) Modulus of a square of the structure factor |*S*(*q*)|^2^ of a linear 1D chain of scatterers with the number *N* = 10 and *N* = 50. (**b**) Calculated 3D image of the diffraction pattern of a 2D photonic structure composed of 10 × 10 elements (for *a*_1_ = *a*_2_ = 1 *μ*m, *λ* = 0.53 *μ*m). (**c**) Calculated and (**d,e**) experimentally measured diffraction patterns from direct (**d**) and inverted (**e**) 2D square photonic structures with the number of scatterers *N*_1_ × *N*_2_ = 5 × 5 observed on a flat screen positioned behind the sample. Insets show the SEM images of the corresponding structures. *a*_1_ = *a*_2_ = 1 *μ*m, *λ* = 0.53 *μ*m.

Now, we analyze a fine structure of the diffraction planes and cones. The function 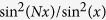 from Eq. (4) in Methods has *N* − 1 zeros between any two adjacent main maxima, and therefore *N* − 2 additional (called ‘subsidiary’ in what follows) maxima. Therefore, we can define the number of scatterers N directly from the experimental diffraction patterns. For conventional 2D photonic films with a large number of scatterers (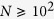), the intensity of the subsidiary maxima is much less or even negligible in comparison with the intensity of the main maxima [Fig. 2(a)]. As a result the cones do not detected when the diffraction is measured from a sample with a big number of scatterers^2^,^3^,^4^. Additionally, the subsidiary maxima located very close to each other, so that any small structural disorder or divergence of a light beam will lead to degradation of a fine structure, and the subsidiary maxima cannot be resolved in the averaged profile of the diffraction patterns [Fig. 1(f,h)]. The entire picture changes dramatically at smaller *N* when the intensity of the subsidiary maxima becomes comparable with the intensity of the main maxima (*N* = 10, Fig. 2(a)), and we obtain a unique chance to observe by eyes the diffraction images registering directly the fine structure in experiment.

**Figure 3:**
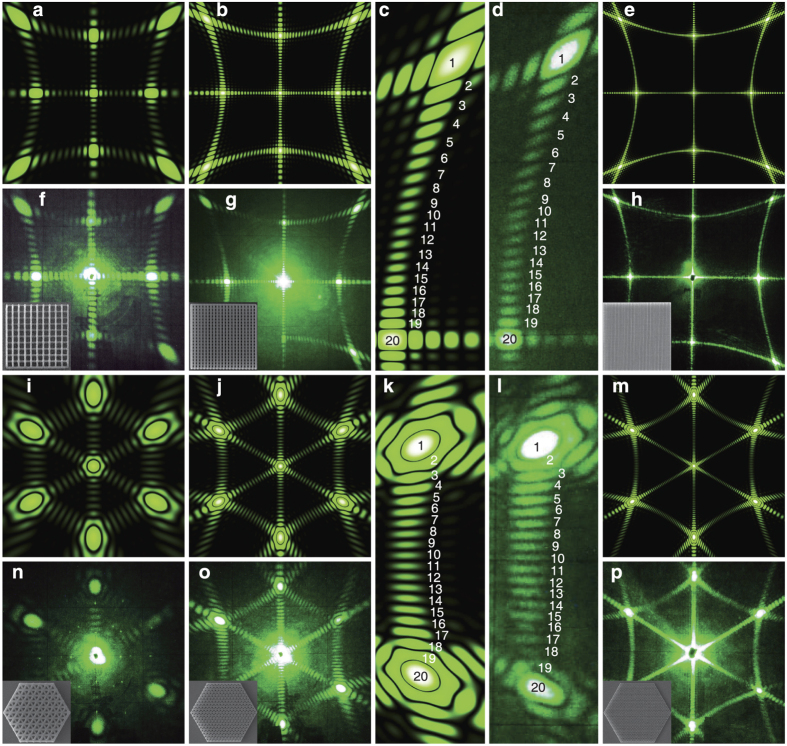
Numerically calculated and corresponding experimentally measured diffraction patterns from 2D square structures with lattice constants *a*_1_ = *a*_2_ = 1 *μ*m (**a–h**) and hexagonal structures with *a* = 1.5 *μ*m (**i–p**). 2D square structures with the number of holes *N*_1_ × *N*_2_ = 10 × 10 (**a,f**), 20 × 20 (**b–d,g**), 50 × 50 (**e,h**). 2D hexagonal structures with the number of forming triangles along the side *N* = 5, (**i,n**), 10 (**j–l,o**), 20 (**m,p**). The patterns observed on a flat screen positioned behind the sample at normal incident beam *λ* = 0.53 *μ*m. Insets show SEM images of the samples.

**Figure 4:**
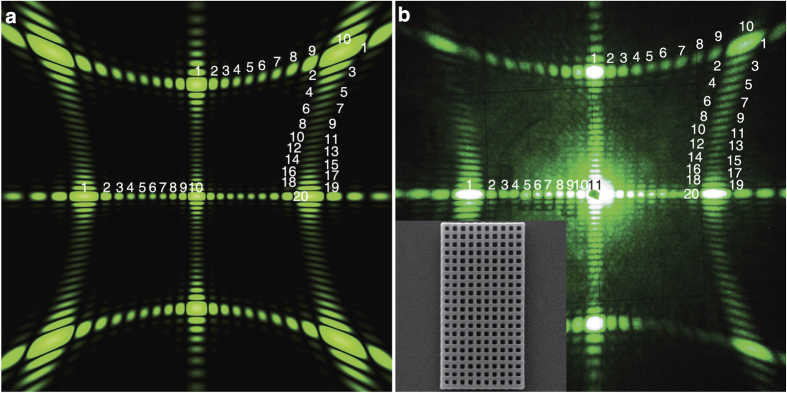
(**a**) Calculated and (**b**) experimentally measured diffraction patterns from rectangular 2D photonic structure *N*_1_ × *N*_2_ = 10 × 20 holes observed on a flat screen positioned behind the sample. Insets show the SEM image of the structure. *a*_1_ = *a*_2_ = 1 *μ*m, *λ* = 0.53 *μ*m.

The results of our experimental studies of light diffraction vs. the size and shape of direct and inverted photonic structures under the normal laser incidence are presented in Figs 2, 3, 4, together with the calculated patterns and SEM imagines of the samples. In experiment, a beam expander is used with an objective for ensuring that the probing laser spot (*λ* = 0.53 *μ*m) is always larger than the overall area of a finite-size photonic structure.

First, we should explicitly identify the type of scatterers for the inverted photonic structures: a hole in the structure or some dielectric elements of the structure scatter light. Figure 2(d,e) show the experimental diffraction patterns from direct 2D structure composed from *N*_1_ × *N*_2_ = 5 × 5 voxels and inverted structure which can be considered either a structure of *N*_1_ × *N*_2_ = 5 × 5 holes or a fishnet – type structure of *N*_1_ × *N*_2_ = 6 × 6 stripes. It is clearly seen that the calculated diffraction pattern for *N*_1_ × *N*_2_ = 5 × 5 scatterers [Fig. 2(c)] and both experimental patterns [Fig. 2(d,e)] are absolutely identical with 3 subsidiary diffraction reflexes between 2 main maxima, 5 reflexes in total. It means that the hole scatters light in 2D inverted dielectric photonic structures.

For all structures with the square symmetry, the diffraction patterns on a flat screen placed after the sample demonstrate the *C*_4*v*_ symmetry at normal incident beam and for all hexagonal structures the diffraction patterns have the *C*_6*v*_ symmetry at normal incidence (Fig. 3). We notice a surprisingly strong intensity of diffraction from a rather small number of submicron dielectric scatterers. For square structures (*a* = 1 *μ*m, *λ* = 0.53 *μ*m, *λ* < *a* < 2*λ*), we can distinguish two types of the diffraction features: (i) two orthogonal (vertical and horizontal) strips that correspond to the zero-order scattering (*n*_1_ = 0 or *n*_2_ = 0); (ii) four arcs that correspond to the first-order scattering (*n*_1_ = 1 or *n*_2_ = 1) being formed by intersections of four cones with a flat screen, as shown schematically in Fig. 1(e). For hexagonal structures (*a* = 1.5 *μ*m, *λ* = 0.53 *μ*m, 2*λ* < *a* < 3*λ*), we can distinguish three types of the diffraction features: (i) three strips (directed at an angle of 60 degrees relative to each other) that correspond to the zero-order scattering; (ii) six arcs that correspond to the first-order scattering and (iii) six another arcs that correspond to the second-order scattering. All arcs are formed by intersections of 6, 12 etc cones with a flat screen, as shown schematically in Methods.

For photonic structures with a large total number of the scatterers (*N*_1_ ⋅ *N*_2_ ~ 10^4^), the experimentally observed strips and arcs look like solid curves with a poorly resolved fine structure only near the main maxima [Fig. 1(f)]. However, with decreasing the number of scatterers *N*^2^, the whole diffraction curves are splitted into isolated reflexes in accord with the theoretical predictions. As a characteristic example, we present the experimental diffraction patterns obtained from direct and inverted photonic structures with different number of scatterers 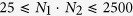 (Figs 2, 3, 4). We observe that for the square photonic structure with 10 × 10 scatterers the arc with fine structure between (0, 1) and (1, 1) main maxima (the notation of the main maxima are shown on Fig. 1(f)) consists of 10 diffraction reflexes (including the two main maxima), for the structure with 20 × 20 scatterers, the arc consists of 20 reflexes [Fig. 3(c) - calculations, Fig. 3(d) – experiment] and so on. For hexagonal structures, the experimentally observed fine structure in arcs between main maxima consist of 2*N* diffraction reflexes (including the two main maxima) in agreement with Eq. (9). Note that for hexagons *N* defines a number of trigonal holes along the side that is the half of the maximal number of trigonal holes 2*N* between two opposite angles of hexagon, as shown in Methods.

For a rectangular structure with 10 × 20 elements, the arc between (0, 1) and (1, 1) consists of 10 reflexes while the arc between (1, 0) and (1, 1) has 20 reflexes (Fig. 4). Note that according to Eq. 3, the apex angle of scattering 2*θ*_*s*_ depends only on lattice parameters *a*_*i*_ in particular direction (for given *λ* and order of scattering *n*). Therefore for the square lattice with *a*_1_ = *a*_2_ four cones produce diffraction patterns with *C*_4*v*_ symmetry. However the numbers of scatterers *N*_1_ and *N*_2_ define the fine structure of the cones and planes that reduce the symmetry of the diffraction patterns to orthogonal *C*_2*v*_. Therefore we can conclude that the general diffraction rules for both square and hexagonal 2D photonic structures are the same and the number of diffraction reflexes is defined by maximal number of scatterers in particular direction.

A specific case is observed experimentally for the zero-order diffraction reflex (0, 0) that coincides with the non-diffractive part of the transmitted laser beam. For this direction, two beams can interfere and, as a result, they produce additional diffraction reflexes thus the total number of experimentally observed reflexes between (0, 0) and the neighboring main maxima (0, 1), (0, −1), (−1, 0), (1, 0) are *N*_1_ + 1 and *N*_2_ + 1, as can be seen from Fig. 4(b) for experimentally observed fine structure in the (−1, 0) − (0, 0) diffraction stripe. This effect is reproduced numerically for the 3D diffraction patterns by using the CST Microwave Studio software [Fig. 2(b)], but it missed in the framework of the Born approximation when only the effects from a sum of single scatterings are evaluated [Fig. 4(a)].

### Variation of diffraction patterns with the sample rotation

**Figure 5:**
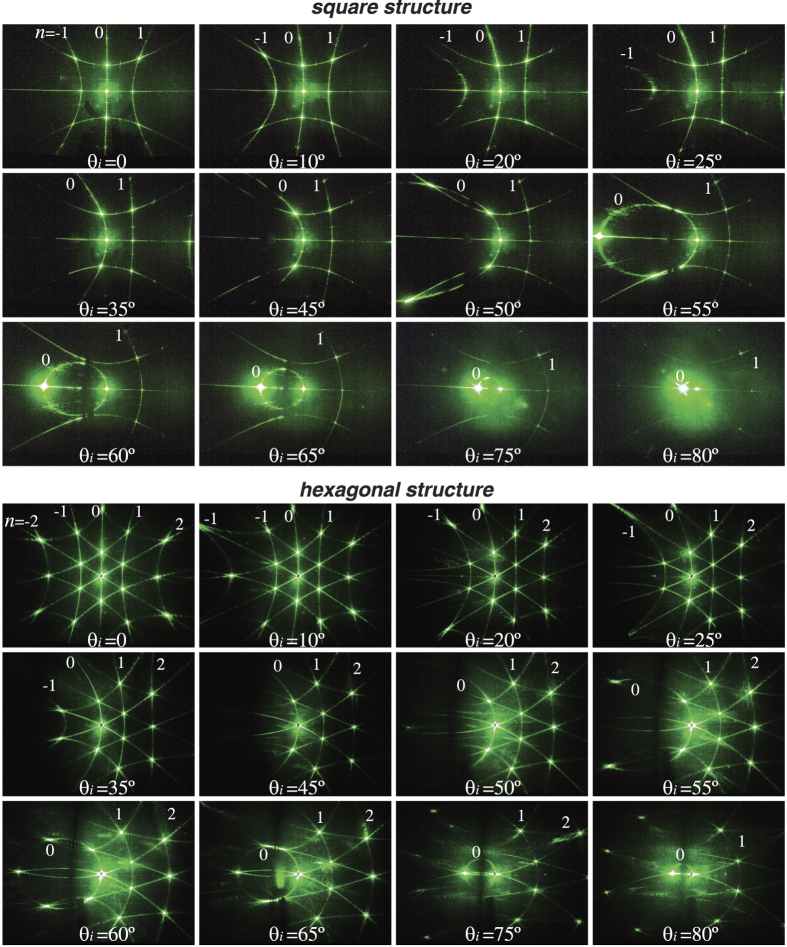
Experimentally recorded transformation of the diffraction patterns for the case of varying the sample rotation angle about vertical axis a_2_ from the normal incidence *θ*_*i*_ = 0 to *θ*_*i*_ = 80°. Twelve upper panels - square sample with *N* = 100, *a*_1_ = *a* = 1 *μ*m, twelve lower panels - hexagonal sample with *N* = 50, *a*_1_ = *a* = 1.5 *μ*m. The patterns are observed on a flat screen positioned behind the sample. *λ* = 0.53 *μ*m.

**Figure 6:**
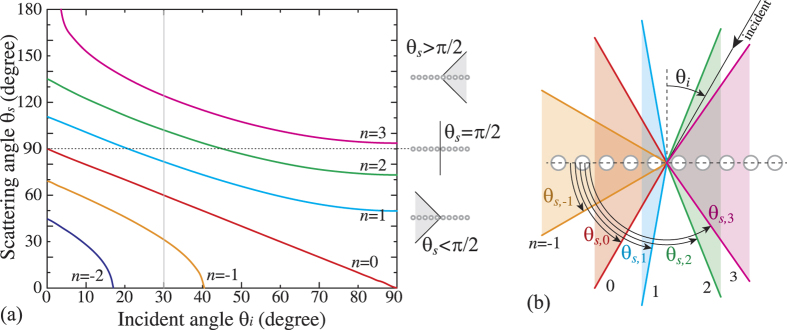
(**a**) Calculated dependencies of the scattering angles *θ*_*s*_ as a function on the incident angle *θ*_*i*_ for different orders of diffraction *n*. (**b**) Schematic of the Laue diffraction from a horizontally oriented chain of scatterers at the incident angle *θ*_*i*_ = 30°. The sketch shows the vertical profiles of five cones for five orders of diffraction, *n* = −1, 0, 1, 2, 3.

Here, we analyze the angular dependence of the diffraction patterns for samples with square and hexagonal symmetry. Figure 5 shows experimental diffraction patterns as the samples are rotated around vertical axis from the normal incidence *θ*_*i*_ = 0 to the angle of *θ*_*i*_ = 80° that is nearly parallel to the laser incident beam. In order to explain the observed effects of appearance, displacement and disappearance of different elements of the diffraction patterns with changing rotation angle *θ*_*i*_, we present in Fig. 6 the calculated scattering angles *θ*_*s*_ as a function on the incident angle *θ*_*i*_ and the order of diffraction *n*. The calculations were performed for horizontal chains of scatterers which are rotated about vertical axis.

The experimental data shows that the transformation of the diffraction patterns from horizontal chains are absolutely identical for square and hexagonal samples. At normal incidence *θ*_*i*_ = 0 the diffraction patterns are symmetrical about vertical axes (Fig. 5). When the samples start to rotate, the diffraction patterns demonstrated several significant changes.

- With *θ*_*i*_ increasing, the curvature of the left arcs (*n*_1_ = −1, −2) increases indicating the decreasing of the apex angle of scattering cones 2*θ*_*s*_, as also seen from Fig. 6. The calculations show that the cone *n*_1_ = −2 collapses (*θ*_*s*_ = 0) at *θ*_*i*_ ≈ 17° while the cone *n*_1_ = −1 collapses at *θ*_*i*_ ≈ 40° in general agreement with experimental data.
- Vertical straight line of zero-order diffraction pattern (*n*_1_ = 0) becomes the arc. It means that the plane of scattered light evolve into the cone with the apex angle 2*θ*_*s*_. The experimental data clearly show that with the sample rotation new circle from zero-order diffraction appears on the flat screen positioned behind the sample. In accordance with the calculations, the apex angle 2*θ*_*s*_ decreases and the cone becomes a horizontal straight line (*θ*_*s*_ = 0) when the laser beam and the chain of scatterers becomes parallel (*θ*_*i*_ = 90°).
- Nontrivial angular dependence is demonstrated by the right arcs (*n*_1_ = +1, +2). Figure 5 shows that with *θ*_*i*_ increasing the curvature of the right arcs decreases indicating the increasing of the apex angle of scattering cones 2*θ*_*s*_. The calculations show that the cone *n*_1_ = +1 evolves into the plane (2*θ*_*s*_ = 180°) at *θ*_*i*_ ≈ 20° while the cone *n*_1_ = +2 evolves into the plane at *θ*_*i*_ ≈ 45° (Fig. 6). With further increase in *θ*_*i*_, both planes evolve back into the cones but with inversed orientation in space as shown in Fig. 6(b) for *θ*_*i*_ = 30°. Indeed, the experimental diffraction patterns for both square and hexagonal structures show that new circles from first- and second-order diffraction (*n*_1_ = +1, +2) appear on the flat screen positioned behind the sample [Fig. 6(a)].

### Transition from photonic crystals to metasurface

It is known that metasurface properties are related to the existence of an effective medium behavior. At the same time we cannot proceed with a homogenization procedure when Bragg diffraction to exist. In this section we analyze a case when Bragg diffraction associated with photonic crystal behavior is suppressed and only zero-order process forms diffraction pattern.

**Figure 7:**
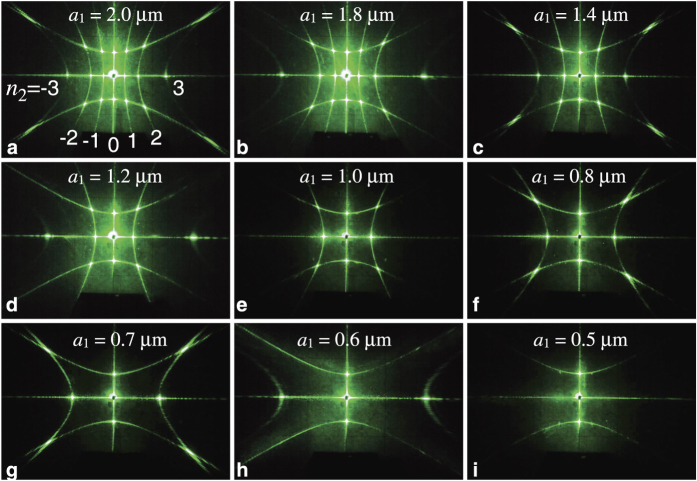
Transition from photonic crystals to metasurfaces. Experimental diffraction patterns obtained from 2D structures with varied lattice parameter 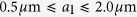 and constant parameter *a*_2_ = 1 *μ*m. Patterns are observed on a flat screen positioned behind the sample. *λ* = 0.53 *μ*m.

The possibility of the experimental observation of a certain order of diffraction *n* is determined by the lattice constant *a* to wavelength *λ* ratio. For variable lattice parameter *a*_1_ and green laser line *λ* = 0.53 *μ*m, the diffraction condition *a*_1_ > |*n*_1_*λ*| allows three pairs of cones (*n*_1_ = ±1, ±2, ±3) for *a*_1_ > 1.59 *μ*m, two pairs of cones (*n*_1_ = ±1, ±2) for 1.59 > *a*_1_ > 1.06 *μ*m and only the first-order diffraction (*n*_1_ = ±1) with one pair of cones for 1.06 > *a*_1_ > 0.53 *μ*m. Figure 7 demonstrates a train of collapses of diffraction cones experimentally observed from a set of 2D square photonic structures with constant lattice parameter *a*_1_ = 1 *μ*m and variable lattice parameter 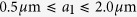. The experimental patterns for the samples with *a*_1_ = 2 and 1.8 *μ*m contain three pares of cones from chains oriented along horizontal axis **a**_1_, two pairs of cones are observed in the cases of *a*_1_ = 1.4 and 1.2 *μ*m and only one pair of cones is observed for *a*_1_ = 1, 0.8, 0.7 *μ*m (Fig. 7). For the sample with *a*_1_ = 0.5 *μ*m < *λ* all these cones are collapsed and only vertical zero-order diffraction plane (*n*_1_ = 0) is observed.

In this experiment, we observe a transition between two regimes of light diffraction, i.e. the regime of Laue diffraction which is characteristic for photonic crystals, and the regime where only the zero-order (*n* = 0) diffraction can be observed under condition *a* < *λ* that is a fingerprint of metasurfaces^22^,^23^,^25^,^26^,^28^,^29^,^35^,^36^. As to the diffraction from chains oriented along vertical axis **a**_2_, the patterns remain unchanged for all samples: one pare of the first-order cones (*n*_2_ = ±1) and horizontal zero-order plane (*n*_2_) are observed.

As clearly seen from Figs 2, 3, 4, 5, 6, 7, the diffraction processes along **a**_1_ and **a**_2_ directions are completely independent for both square and rectangular samples that are quite natural for low-contrast photonic structures^7^. Note that in these structures one can identify another families of chains of scatterers including and with the highest priority the diagonal chains **a**_1_ ± **a**_2_. However, we can not observe experimentally or in simulations any traces of the characteristic diffraction patterns along **a**_1_ ± **a**_2_ directions even for the photonic structures with high number of scatterers [Fig. 1(f)]. And this despite the fact that the zero-order diffraction planes (*n* = 0) oriented at the diagonal angles of ±45° to the **a**_1,2_ directions should be observed at any ratio between *λ* and **a**_1,2_. This effect differs fundamentally from 3D light diffraction observed for example in synthetic opals where different {*hkl*} crystal planes determine the 3D Bragg diffraction patterns^37^.

## Discussion

Our experimental and theoretical studies have shown that 2D photonic structures reveal many remarkable optical effects. By specially, choosing the lattice parameters and laser wavelength, we have visualized the diffraction features for both direct and inverted 2D structures on a flat screen placed behind the sample. We have observed experimentally a fine structure of the diffraction from finite-size 2D dielectric structures that provides not only information about the structure symmetry but also allows characterizing the shape and determining exactly the number of scatterers. When *N*_1_ or *N*_2_ increases, isolated reflexes start to overlap and finally merge into continuous diffraction patterns similar to the general merging of isolated energy levels into continuous bands in crystalline structures. The symmetry of continuous diffraction patterns is defined by the symmetry of the 2D lattice but the number of isolated reflexes is defined by the maximal number of scatterers (*N*_1_, *N*_2_) in particular direction. Therefore the exact symmetry of fine structure in diffraction patterns is defined by the shape of the 2D sample. Taking advantage of the Laue diffraction independency in different orientations, we present an elegant way to demonstrate the transition in the light regimes between 2D photonic structure and metasurface. Our theory is in very good agreement with experiment.

It was found both theoretically and experimentally that the diffraction patterns at different high symmetry directions are independent. As a result a set of anisotropic samples with orthogonal *C*_2*v*_ lattice symmetry, variable lattice parameter along **a**_1_ and unchangeable lattice parameter along **a**_2_, demonstrates unvarying diffraction patterns in one direction and transition from Laue diffraction typical for photonic crystals to a non-diffraction regime characteristic for metamaterials in other direction.

The 3D diffraction pattern obtained for the photonic structure with small number of scatterers can be considered as radiation patterns of an optical antenna with spatially-resolved lobes [Fig. 2(b)]. The photonic structure converses a laser beam into several sets of high-directive lobes and moulds wavefront in exact correspondence with the number and arrangement of the dielectric scatterers. This property gives a promise for applications of 2D finite-size photonic structures as a superdirective all-dielectric metaantennas. Each antenna’s lobe corresponds to the maxima of the structure factor |*S*(**q**)|^2^. We notice that the similar scattering problem is known as *N*-element RF-antenna array^38^.

## Methods

### Diffraction from two-dimensional periodic structures

#### Square and rectangular structures

For the 2D photonic structures with the square and orthogonal lattice symmetry, the position of each scatterer (voxels or hole) is determined by the 2D vector **r**_*i*_ = **a**_1_*n*_1_ + **a**_2_*n*_2_, where **a**_1_ and **a**_2_ stand for the basis mutually orthogonal vectors (**a**_1_ ⋅ **a**_2_ = 0) of the square (*a*_1_ = *a*_2_) or rectangular (*a*_1_ ≠ *a*_2_) lattice, 0 ≤ *n*_*j*_ ≤ *N*_*j*_ and *N*_*j*_ are integer.

For the analysis of diffraction patterns from low-contrast periodic structures (that include the photonic structures fabricated by the DLW technique), it is usually sufficient to use the Born approximation when the interaction between the scatterers is neglected^39^. In the Born approximation, the diffraction intensity is determined by a product of the squares of the structure factor *S*(**q**), which is associated with the lattice periodicity, the scattering form factor *F*(**q**), which takes into account the contribution from a unit cell, and a polarization factor. A comparison of the theoretical and experimental data shows that in our case of the quasi-point scatterers it is sufficient to consider only the structure factor *S*(**q**). Under these conditions, the diffraction angles and peak intensities become simple functions of the crystallographic 2D structure^40^:


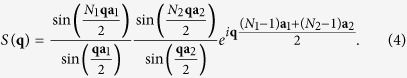


Here **q** = **k**_*i*_ − **k**_*s*_ is the scattering vector, whereas **k**_*i*_ and **k**_*s*_ are the wave vectors of the incident and scattered waves. Thus, the diffraction patterns depend either on the size of the sample if the whole sample is illuminated or on the number of illuminated scatterers *N*_*j*_ along the directions of the vectors **a**_1_ and **a**_2_, respectively. Equation (4) is valid for a structure with a parallelogram shape and for the square and rectangle as the special cases of the parallelogram^40^.

#### Hexagonal structures

**Figure 8:**
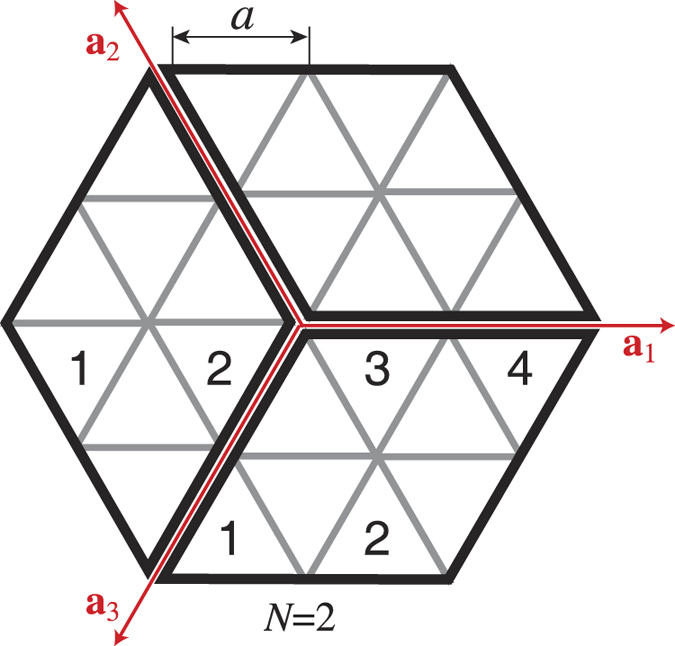
Schematic of the hexagon’s subdivision into three parallelograms. The length of the hexagon side equals to *Na*. The maximal size of the hexagon (its diagonal) equals to 2*Na*. **a**_*j*_ are the basis vectors of the hexagonal lattice.

To analyze the diffraction from the 2D photonic structures with the hexagonal symmetry *C*_6*v*_ it is convenient to consider three basis vectors **a**_1_, **a**_2_ and **a**_3_ instead of two vectors because all three directions in the lattice are equivalent (Fig. 8). To calculate the structural factor *S*(**q**), we subdivide the hexagon into three parallelograms forming by three pairs of vectors (**a**_1_, **a**_2_), (**a**_2_, **a**_3_) and (**a**_3_, **a**_1_), as shown in Fig. 8. The structural factor *S*_*ij*_(**q**) for each parallelogram is calculated from Eq. (4) and can be written as:


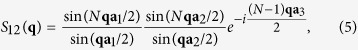



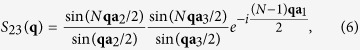



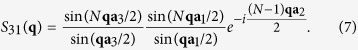


When dealing with the whole hexagon, one should combine three Eqs (5, 6, 7) taking into account the origin of coordinates for all parallelograms, to obtain:


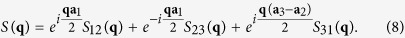


It is straightforward to demonstrate that the square of the structure factor modulus can be rewritten as:


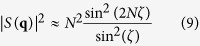


where 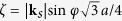, *φ* is an angle between **k**_*s*_ and **k**_*i*_. The *x*-component of **k**_*s*_ is assumed to be zero. In our numerical calculations a hexagon is given by the lattice constant *a* and by the number of forming triangles *N* (Fig. 8).

## Additional Information

**How to cite this article**: Rybin, M. V. *et al.* Transition from two-dimensional photonic crystals to dielectric metasurfaces in the optical diffraction with a fine structure. *Sci. Rep.* **6**, 30773; doi: 10.1038/srep30773 (2016).
